# Emotional Functioning as a Dimension of Quality of Life in Breast Cancer Survivors: A Systematic Review and Meta-Analysis

**DOI:** 10.3390/cancers17223707

**Published:** 2025-11-19

**Authors:** Iryna Makhnevych, Mussab Ibrahim Mohamed Fadl Elseed, Ibrahim Mohamed Ahmed Musa, Yauhen Statsenko

**Affiliations:** 1 Psychiatry Department, Cleveland Clinic Abu Dhabi, Abu Dhabi P.O. Box 112412, United Arab Emirates; 2 Department of Radiology, College of Medicine and Health Sciences, United Arab Emirates University, Abu Dhabi P.O. Box 15551, United Arab Emirates; musabibrahim@uaeu.ac.ae (M.I.M.F.E.); hema2030hema@gmail.com (I.M.A.M.)

**Keywords:** breast cancer, emotional functioning, quality of life, survivorship, meta-analysis, surgery, age differences, trajectories, psychosocial support

## Abstract

**Background**: As survival rates among breast cancer (BC) patients continue to rise, Emotional Functioning (EF)—has become increasingly clinically relevant; however, researchers have yet to fully characterize its long-term, dynamic trajectories following surgery. This systematic review and meta-analysis aimed to (1) characterize the longitudinal trajectories of EF after BC surgery and (2) examine the moderating effects of surgical modality and age. **Methods**: We conducted this systematic review and meta-analysis in accordance with PRISMA 2020 guidelines. We synthesized data from studies published between 2000 and 2024 that assessed EF using the EORTC QLQ-C30 at multiple post-surgical time points. Using multilevel random-effects meta-analytic models, we examined EF trajectories across 116 effect sizes derived from 40 studies, and evaluated time, surgical modality (breast-conserving surgery (BCS), mastectomy (MA), mastectomy with immediate reconstruction (Mx + IR) and age group as moderators. **Results**: The overall pooled estimate for EF was 73.44 (95% CI: 70.29–76.58, *p* < 0.001). Time since surgery significantly influenced EF: scores were lowest during the initial 6 months (66.82, 95% CI: 59.75–73.89), peaked at 7–15 months (77.86, 95% CI: 74.51–81.22) and 31–54 months (77.52, 95% CI: 70.44–84.59), and showed lower values at 16–30 months (72.58, 95% CI: 61.45–83.72) and 55–72 months (69.81, 95% CI: 64.08–75.54). Surgical modality significantly shaped these trajectories (*p* = 0.013). The overall pooled estimate for EF was 73.44 (95% CI: 70.29–76.58, *p* < 0.001). Time since surgery significantly influenced EF: scores were lowest during the initial 6 months (66.82, 95% CI: 59.75–73.89), peaked at 7–15 months (77.86, 95% CI: 74.51–81.22) and 31–54 months (77.52, 95% CI: 70.44–84.59), and showed lower values at 16–30 months (72.58, 95% CI: 61.45–83.72) and 55–72 months (69.81, 95% CI: 64.08–75.54). Surgical modality significantly shaped these trajectories (*p* = 0.013). The BCS group showed a significant inverted-U trajectory in EF scores, with a positive linear slope (β = 1.22, SE = 0.50, *p* = 0.046) and a small negative quadratic term (β = −0.02, SE = 0.01, *p* = 0.046), indicating initial improvement followed by decline. A similar pattern was observed for MA, where the linear term (β = 1.19, SE = 0.51, *p* = 0.054) and quadratic curvature (β = −0.02, SE = 0.01, *p* = 0.054) suggested an early rise with subsequent decline. In contrast, Mx + IR displayed a high intercept (β = 71.46, SE = 4.46, *p* < 0.001) but no significant trajectory over time (*p* = 0.582), indicating stability. The 45–60 year group demonstrated a significant inverted-U trajectory in EF scores, with a positive linear coefficient (β = 0.87, SE = 0.38, *p* = 0.067) and a negative quadratic coefficient (β = −0.01, SE = 0.01, *p* = 0.067), suggesting an early rise in emotional functioning followed by a subsequent decline. Participants <45 years also showed a significant inverted-U pattern, starting from a moderately high baseline (β = 67.56, SE = 4.26, *p* < 0.001) with a positive linear slope (β = 0.82, SE = 0.34, *p* = 0.051) and a negative quadratic curvature (β = −0.01, SE = 0.01, *p* = 0.051). In contrast, the >60 year group reported the highest baseline scores (β = 75.60, SE = 5.18, *p* < 0.001) with no significant trajectory, indicating overall stability. These findings confirm that EF follows a significant inverted-U trajectory (*p* < 0.001) and is influenced by time, surgical modality, and age.

## Simple Summary

In this systematic review and meta-analysis, we examined the long-term trajectories of Emotional Functioning (EF)—a core dimension of quality of life (QoL)—in breast cancer (BC) survivors following surgery. We synthesized data from 40 studies published between 2000 and 2024, comprising 116 observations of EF measured by the EORTC QLQ-C30. The analysis revealed a significant inverted-U trajectory in EF, showing an initial improvement during the first 24–30 months after surgery, followed by a gradual decline toward baseline levels by 60 months. Surgical modality significantly shaped these trajectories: breast-conserving surgery (BCS) and mastectomy (MA) produced similar inverted-U patterns, whereas mastectomy with immediate reconstruction (Mx + IR) sustained stable EF over time. Age further influenced trajectories, with younger and middle-aged survivors experiencing early gains but subsequent declines. Findings underscore the dynamic nature of EF and highlight the need for sustained psychosocial support, particularly for middle-aged women and those undergoing mastectomy, to promote long-term emotional well-being.

## 1. Introduction

BC is among the most prevalent malignancies affecting women worldwide, and due to substantial progress in early detection and treatment, survival rates have markedly improved over recent decades [1]. As survival outcomes continue to rise, QoL has emerged as a critical dimension of survivorship research and clinical care [2]. Within this multidimensional construct, EF represents a central yet often under-examined domain [2,3]. EF captures several interrelated aspects of emotional life, including the ways individuals express, interpret, comprehend, respond to, and manage their emotions [4]. In the context of BC, EF further encompasses affective well-being, emotional distress, fear of cancer recurrence, body image concerns, and the individual’s capacity for psychological adjustment throughout survivorship [2]. Gaining a deeper understanding of EF trajectories is crucial for promoting comprehensive recovery and sustaining long-term QoL among BC survivors.

Growing evidence suggests that EF is not static but evolves dynamically over time following surgery, often following nonlinear trajectories shaped by surgical modality, age, and broader biopsychosocial factors [3,5,6,7]. For instance, a prospective cohort study in the Netherlands demonstrated that while EF tends to improve progressively after surgery, psychosocial well-being may decline—particularly among women who do not undergo reconstruction [3]. Among BC patients, QoL follows a curvilinear trajectory, showing initial postoperative improvements that reach a peak around 31 to 54 months, after which it either stabilizes or gradually declines [7].

Research underscores the heterogeneity of emotional adjustment among BC survivors [3,8]. Studies among older survivors have delineated distinct depressive symptom trajectories, revealing that although most patients maintain stable emotional profiles, a subset exhibits delayed recovery or increasing symptomatology over a three-year period—patterns strongly associated with lower social support and higher baseline anxiety [6]. Insufficient family support has been associated with poorer psychosocial well-being trajectories during the first postoperative year among breast cancer survivors [3].

Surgical modality also appears to influence emotional adjustment, with evidence suggesting that women undergoing breast reconstruction may experience more rapid improvements in EF compared with those receiving MA or BCS [3]. Moreover, data indicate that younger (<50 years) women often report higher emotional distress compared to older survivors [9]. Together, these studies suggest that EF trajectories are shaped by both treatment type and age-related psychosocial vulnerabilities. These findings highlight the need to examine EF longitudinally and to account for the influence of clinical and contextual factors across time.

Despite this emerging body of literature, critical knowledge gaps remain. Notably, few studies have synthesized longitudinal data to model the long-term, potentially nonlinear trajectories of EF beyond the initial 2–3 years after surgery [2,5,6,7]. Moreover, the moderating roles of surgical modality and age have not been comprehensively quantified across studies [3,7]. Addressing these gaps is essential for identifying periods of heightened emotional vulnerability, informing the timing and content of psychosocial interventions, and optimizing survivorship care strategies tailored to individual needs.

Understanding EF trajectories also holds significant clinical implications. Persistent emotional distress has been linked to poorer adherence to adjuvant therapies, increased healthcare utilization, and reduced overall well-being [10,11]. Integrating EF monitoring into survivorship care pathways may therefore enhance holistic recovery and long-term adaptation. A meta-analytic approach that synthesizes existing longitudinal evidence offers an opportunity to clarify these trajectories, uncover heterogeneity, and guide evidence-based, age- and treatment-specific psychosocial interventions.

Accordingly, this systematic review and meta-analysis sought to comprehensively synthesize longitudinal evidence on EF among BC survivors following surgical treatment. Specifically, the objectives were to: (1) characterize trajectories of EF over time after surgery, including potential nonlinear patterns; and (2) examine the moderating effects of surgical modality and age on these trajectories.

## 2. Materials and Methods

### 2.1. Study Design and Eligibility Criteria

The methodological approach used in this study follows the framework previously described in our earlier publication [7] with appropriate adaptations for the current research context. We conducted this systematic review and meta-analysis following the PRISMA reporting framework [12]. The review has been registered in the International Prospective Register of Systematic Reviews (PROSPERO) under the identification number CRD42024565182. We performed a comprehensive literature search across major biomedical databases—Scopus, CINAHL, Embase, APA PsycArticles, PubMed, SciELO, LILACS, and the Global Index Medicus—to identify eligible publications. Supplementary Table S3 provides the complete search strategy and parameters.

The review covered original studies that assessed QoL among BC patients after surgical treatment, used validated instruments such as the EORTC QLQ-C30, and reported sufficient statistical information (means, standard deviations, and sample sizes) to calculate effect sizes at multiple postoperative time points. We limited our search to studies published between 2000 and 2024. To ensure methodological consistency, we excluded protocol papers, letters, conference abstracts, and studies that did not report EF/QoL outcomes or that used alternative QoL measures.

All retrieved records were imported into the Covidence systematic review management platform [13] for automated deduplication and screening. Three reviewers [I.M., M.E., I.M.A.M.] independently screened titles and abstracts using predefined inclusion and exclusion criteria. When disagreements arose, the reviewers resolved them through discussion with the principal investigator. We applied the same process to full-text screening, documented reasons for exclusion, and summarized the selection process in the PRISMA flow diagram (Figure S1, Supplementary Materials). 

### 2.2. Data Extraction and Quality Appraisal

Using a standardized data extraction template, we independently collected detailed information on study design, participant characteristics, methodological elements, and main findings (EF) [I.M., M.E., I.M.A.M.]. Our team assessed the methodological quality and potential risk of bias for each included study using the Joanna Briggs Institute (JBI) critical appraisal tool [14,15]. Supplementary Tables S1 and S2 summarize the extracted effect measures and quality assessments. 

### 2.3. Data Compilation and Coding

The final dataset included 116 effect size estimates from studies using the EORTC QLQ-C30 questionnaire [16,17,18,19,20,21,22,23,24,25,26,27,28,29,30,31,32,33,34,35,36,37,38,39,40,41,42,43,44,45,46,47,48,49,50,51,52,53,54,55]. For each study, we extracted identifiers, patient age categories (<45, 45–60, >60 years), surgical modality (mastectomy [MA], breast-conserving surgery [BCS], mastectomy with immediate reconstruction [Mx + IR], or other procedures), measurement instrument, postoperative time points, mean EF scores, standard deviations, and sample sizes. EF scores range from 0 to 100, with higher values indicating better QoL. We grouped all immediate reconstruction procedures after mastectomy into the “Mx + IR” category because limited reporting and study heterogeneity prevented stratification by reconstruction technique. Implant-based and autologous reconstructions may influence QoL differently. Postoperative time points as follows: 0–6 months (coded as 3), 7–15 months (12), 16–30 months (24), 31–54 months (48), 55–72 months (60), and >73 months (120). When available, we retained the original reported time values. 

### 2.4. Statistical Analysis

All statistical analyses were conducted using R software (version 4.4.1) [56]. Our analysis used multilevel random-effects models to handle both within- and between-study heterogeneity. To model temporal trajectories, we resorted to linear, quadratic, and logarithmic functions and evaluated model fit using the Akaike Information Criterion (AIC) and Bayesian Information Criterion (BIC) [57]. EF trajectories were stable high, stable moderate, persistently low, improving, declining, U-, or inverted-U-shaped, which was reflected in regression coefficients. We calculated effect sizes as unstandardized mean differences and derived corresponding variances with 95% confidence intervals using standard meta-analytic procedures. Each variance was computed as the squared standard deviation of the harmonized score divided by its sample size. To quantify between-study heterogeneity, we employed three indices: (1) Cochran’s Q statistic to test the null hypothesis of homogeneity [58]; (2) the I^2^ statistic to estimate the proportion of total variation due to heterogeneity [59]; and (3) τ^2^ (tau-squared) as the measure of between-study variance. We interpreted I^2^ values around 25%, 50%, and 75% as low, moderate, and high heterogeneity, respectively [60]. 

### 2.5. Subgroup, Meta-Regression, and Bias Analyses

We examined EF trajectories across subgroups defined by surgical type, patient age group, and postoperative time period. With meta-regression analyses our team evaluated time as a continuous moderator and tested interaction effects between surgical type and age group. We considered results statistically significant at α = 0.05. Publication bias was assessed visually with funnel plots and statistically with Egger’s regression test [61]. We conducted a leave-one-out sensitivity analysis to examine the robustness of the findings. 

### 2.6. Visualization and Ethical Considerations

Forest plots and trajectory curves helped us to visualize EF patterns across time points, surgical categories, and age groups. We weighted each study estimate according to its sample size and presented 95% confidence intervals. Because this study did not involve human or animal subjects, it did not require formal ethical approval. All materials—including data extraction templates, analysis code, and protocols—are available from the corresponding author upon reasonable request.

## 3. Results

### 3.1. Emotional Health

#### 3.1.1. Descriptive Statistics

The EF analysis included 40 studies contributing 116 effect sizes from the EORTC QLQ-C30 instrument. Multiple effect sizes per study occurred when: (1) studies reported EF at multiple follow-up time points (e.g., at 3, 12, and 24 months post-surgery), (2) studies included multiple treatment arms compared to a control group, or (3) studies stratified results by clinically relevant subgroups such as cancer stage or treatment type. This approach maximizes data utilization from each study while our multilevel meta-analytic model accounts for the non-independence of multiple observations from the same study through nested random effects (effect sizes nested within studies).

Across the 40 studies included, 29 studies recruited participants with BC stages 0–III, 7 studies included participants with stages 0–IV, and 4 studies did not clearly specify cancer stage information. Regarding surgical interventions, the largest subgroup comprised patients who underwent MA (38.8%, *n* = 45), followed by BCS (35.3%, *n* = 41) and Mx + IR (22.4%, *n* = 26). A smaller portion received other procedures (3.5%, *n* = 4). In terms of time since surgery, most patients were assessed within the first 6 months postoperatively (29.3%, *n* = 34) or between 7 and 15 months (29.3%, *n* = 34). Fewer data points came from longer-term follow-ups (16–30 months: 8.6%, *n* = 10; 31–54 months: 8.6%, *n* = 10; and 55–72 months: 7.8%, *n* = 9), while 16.4% (*n* = 19) had unknown follow-up times. For age groups, the majority of patients were 45–60 years (68.1%, *n* = 79), with smaller proportions <45 years (9.5%, *n* = 11), >60 years (6.9%, *n* = 8), and unknown age (15.5%, *n* = 18).

#### 3.1.2. Subgroup Analysis of Emotional Functioning

**Figure 1:**
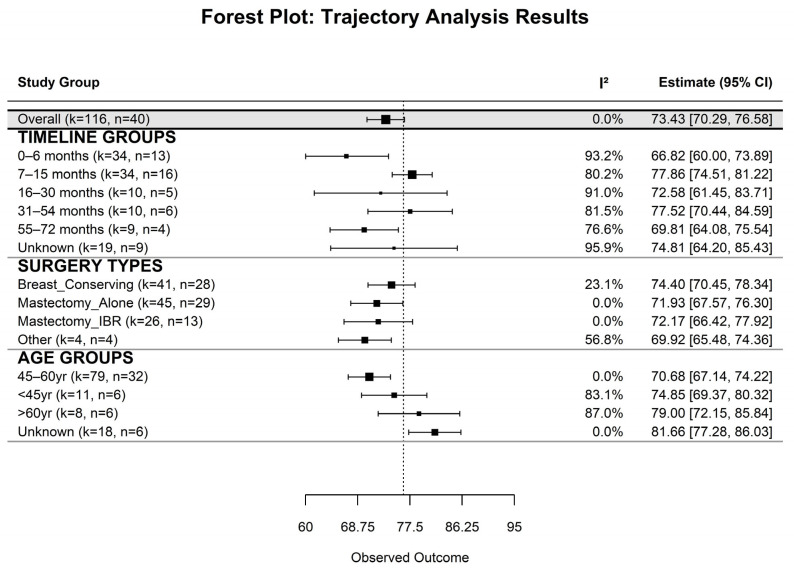
Forest Plot of EF by Timeline, Surgery Type, and Age Group. Each point represents the pooled mean EF score (with 95% CI) from meta-analyses across subgroups. I^2^ indicates heterogeneity. “k” denotes the number of effect sizes; “*n*” is the total sample within each subgroup.

The pooled analysis revealed significant variations in EF among BC survivors following surgery, influenced by time since surgery, surgical modality, and age group. The overall pooled estimate for EF was 73.44 (SE = 1.61, 95% CI: 70.29–76.58, *p* < 0.001), with no between-study heterogeneity (I^2^ = 0%) (Table A1, Figure 1 and Figure A2). Time since surgery significantly influenced QoL: scores were lowest during the initial 6 months (66.82, 95% CI: 59.75–73.89, I^2^ = 93.2%), peaked at 7–15 months (77.86, 95% CI: 74.51–81.22, I^2^ = 80.2%) and 31–54 months (77.52, 95% CI: 70.44–84.59, I^2^ = 81.5%), and showed lower values at 16–30 months (72.58, 95% CI: 61.45–83.72, I^2^ = 91.0%) and 55–72 months (69.81, 95% CI: 64.08–75.54, I^2^ = 76.6%). Variability remained high across most timepoints.

Across surgical modalities, the highest EF was observed in the BCS group (74.40, 95% CI: 70.45–78.34, I^2^ = 23.1%), while MA group (71.93, 95% CI: 67.57–76.30, I^2^ = 0%), Mx + IR (72.17, 95% CI: 66.42–77.92, I^2^ = 0%), and other procedures (69.92, 95% CI: 65.48–74.36, I^2^ = 56.8%) yielded lower scores. For age groups, EF was highest among participants of unknown age (81.66, 95% CI: 77.28–86.03, I^2^ = 0%) and those >60 years (79.00, 95% CI: 72.15–85.84, I^2^ = 87.0%). Patients aged <45 years reported a mean of 74.85 (95% CI: 69.37–80.32, I^2^ = 83.1%), while those aged 45–60 years had lower scores (70.68, 95% CI: 67.14–74.22, I^2^ = 0%).

#### 3.1.3. Trajectory Modeling for Emotional Functioning

To examine the longitudinal pattern of EF following BC surgery, multiple trajectory models were tested and the best-fitting functional form was identified.

Among the tested models for EF trajectory, the quadratic model demonstrated the best fit, with the lowest AIC (10977.48) and BIC (10994.90) values (Table A2). While all models explained the same amount of variance (R^2^ = 1.000), the quadratic model outperformed the logarithmic and linear alternatives by substantial margins (ΔAIC = 13.90 and 20.26, respectively). These differences in fit statistics support the selection of a quadratic model, consistent with the observed inverted-U trajectory where emotional QoL initially improves, then gradually declines over time.

**Figure 2:**
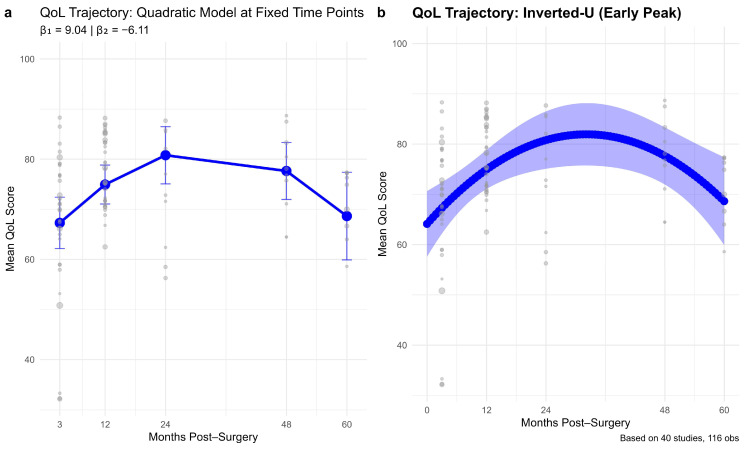
Trajectory of EF over 60 months following surgery.

The left plot displays pooled mean EF scores at fixed time intervals (~3, 12, 24, 48, and 60 months post-surgery) (Figure 2). QoL increased from approximately 67.3 at 3 months (95% CI: 62.2–72.4) to 74.9 at 12 months (95% CI: 71.1–78.8), reaching a peak of 80.8 at 24 months (95% CI: 75.1–86.5). Scores then declined to 77.6 at 48 months (95% CI: 72.0–83.3) and further to 68.6 by 60 months (95% CI: 59.9–77.4). This pattern suggests an early post-surgical improvement, followed by a gradual deterioration over time.

The right panel illustrates the predicted emotional QoL trajectory using a quadratic model fitted to continuous follow-up time. The model revealed a classic inverted-U shape, with a positive linear effect (B = 9.04, *p* = 0.0015) and a significant negative quadratic curvature (B = −6.11, *p* = 0.0019). Scores rose steadily until ~24–30 months post-surgery, where peak predicted QoL approached ~81, followed by a gradual decline to approximately 69–70 by 60 months.

#### 3.1.4. Emotional Functioning Trajectory Analysis by Surgical Modality

**Figure 3:**
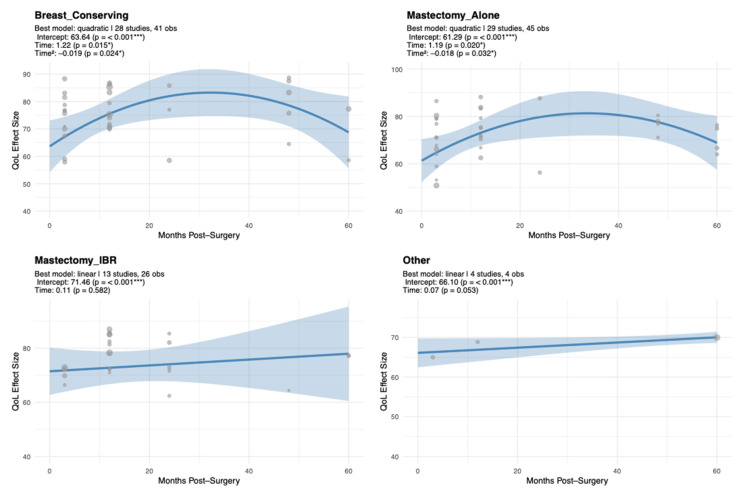
Subgroup EF Trajectories by Surgery Type (Unadjusted Models). Each panel represents a separate unadjusted meta-regression conducted within the respective surgery type. Models were fitted independently and not adjusted for age, baseline EF, or other study-level moderators. Time represents months post-surgery (linear term); Time^2^ represents the quadratic term. Significance codes: *** *p* < 0.001, * *p* < 0.05.

EF trajectories exhibited significant variation according to surgical type, revealing dynamic patterns over time. EF outcomes varied by surgical type. The BCS group showed a significant inverted-U trajectory in EF scores, with a positive linear slope (β = 1.22, SE = 0.50, *p* = 0.046) and a small negative quadratic term (β = −0.02, SE = 0.01, *p* = 0.046), indicating initial improvement followed by decline (Figure 3, Table A3).

A similar pattern was observed for MA, where the linear term (β = 1.19, SE = 0.51, *p* = 0.054) and quadratic curvature (β = −0.02, SE = 0.01, *p* = 0.054) suggested an early rise with subsequent decline. In contrast, Mx + IR displayed a high intercept (β = 71.46, SE = 4.46, *p* < 0.001) but no significant trajectory over time (*p* = 0.582), indicating stability. The other surgery group also showed a stable profile, with no significant slope (β = 0.07, SE = 0.03, *p* = 0.053).

#### 3.1.5. Trajectory Model Estimates for Emotional Functioning by Age

**Figure 4:**
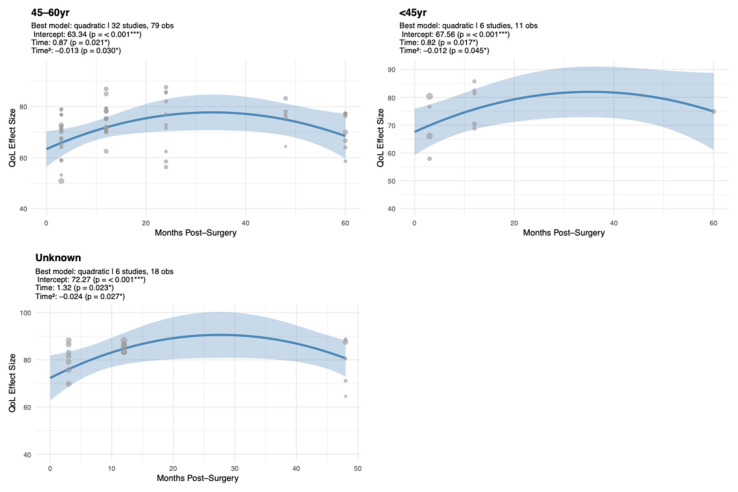
Unadjusted Meta-Regression of EF Scores Over Time by Age Group. Each panel represents a separate unadjusted meta-regression conducted within the respective surgery type. Models were fitted independently and not adjusted for age, baseline EF, or other study-level moderators. Time represents months post-surgery (linear term); Time^2^ represents the quadratic term. Significance codes: *** *p* < 0.001, * *p* < 0.05.

EF trajectories differed by age, with each group displaying distinct patterns of stability and change over time. The 45–60 year group demonstrated a significant inverted-U trajectory in EF scores, with a positive linear coefficient (β = 0.87, SE = 0.38, *p* = 0.067) and a negative quadratic coefficient (β = −0.01, SE = 0.01, *p* = 0.067), suggesting an early rise in emotional functioning followed by a subsequent decline (Table A4; Figure 4). Participants <45 years also showed a significant inverted-U pattern, starting from a moderately high baseline (β = 67.56, SE = 4.26, *p* < 0.001) with a positive linear slope (β = 0.82, SE = 0.34, *p* = 0.051) and a negative quadratic curvature (β = −0.01, SE = 0.01, *p* = 0.051). In contrast, the >60 year group reported the highest baseline scores (β = 75.60, SE = 5.18, *p* < 0.001) with no significant trajectory, indicating overall stability. For individuals with unknown age, the trajectory displayed a significant quadratic profile, with a positive linear effect (β = 1.32, SE = 0.58, *p* = 0.075) and a negative quadratic term (β = −0.02, SE = 0.01, *p* = 0.075), indicating initial improvement followed by later decline.

#### 3.1.6. Meta Regression for Emotional Functioning

This model used patients younger than 45 years who underwent BCS as the baseline group to examine how age and surgical type influenced emotional health trajectories.

EF followed a significant inverted-U trajectory, with a positive linear slope (B = 11.51, *p* = 0.0004) and a negative quadratic curvature (B = −5.37, *p* = 0.013) (Table 1).

Relative to baseline, patients aged 45–60 years (B = 2.30, *p* = 0.109) and those >60 years (B = 0.74, *p* = 0.748) did not differ significantly in overall EF, while individuals of unknown age reported significantly higher scores (B = 7.28, *p* = 0.008). Compared with BCS, MA was associated with significantly lower EF (B = −1.87, *p* = 0.022), while Mx + IR (B = −2.97, *p* = 0.117) and other procedures (B = −6.24, *p* = 0.188) were not significantly different.

Age and time interacted in important ways. EF of patients aged 45–60 years showed a significant negative interaction with time (B = −4.64, *p* < 0.001), indicating a flatter or declining trajectory compared to baseline. EF of those of unknown age also trended toward a decline over time (B = −4.15, *p* = 0.156), though not significantly. Interactions between surgery type and time dynamics in EF were mostly non-significant, though “other” procedures suggested a possible sharper decline over time (B = −9.65, *p* = 0.079) with a compensatory positive quadratic effect (B = 6.02, *p* = 0.089).

The omnibus test revealed that time points (χ^2^ = 13.419, df = 4, *p* = 0.009), surgery types (χ^2^ = 11.264, df = 3, *p* = 0.010), and age groups (χ^2^ = 15.318, df = 3, *p* = 0.002) significantly moderated EF, indicating that these factors accounted for meaningful heterogeneity across studies (Table A5). In contrast, study design (χ^2^ = 2.818, df = 4, *p* = 0.589) was not a significant moderator, suggesting that it did not explain variability in EF outcomes.

**Figure A1:**
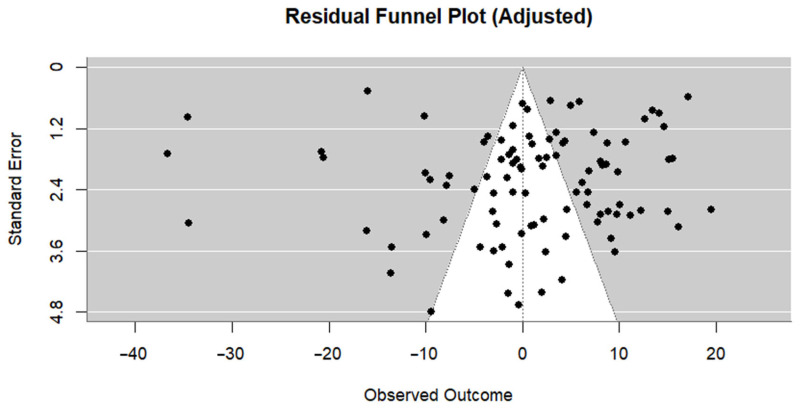
Publication bias.

**Figure A2:**
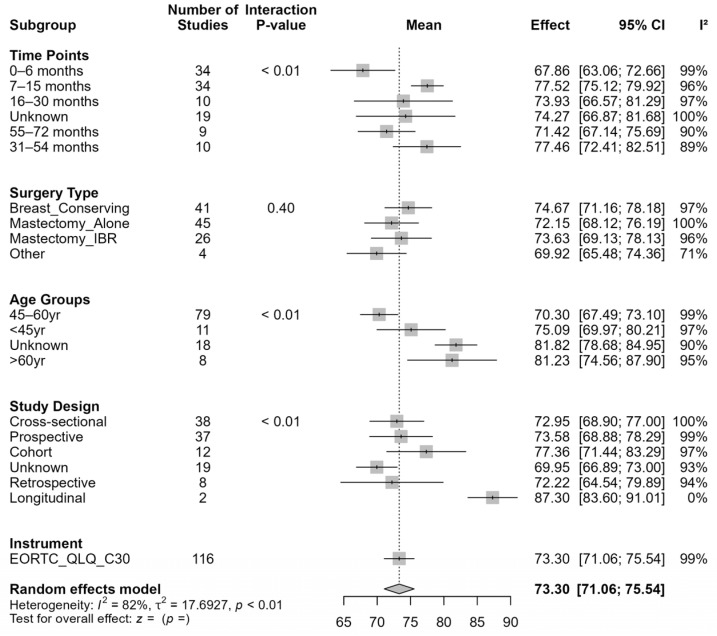
Subgroup analysis results. Pooled EF scores are harmonized on a 0–100 scale; higher scores indicate better EF. Random-effects models were used with REML estimation. I^2^ reflects the proportion of variance due to heterogeneity. *p* values indicate whether subgroup differences significantly explain heterogeneity.

Egger’s regression test was performed by regressing residuals on precision (Figure A1). The slope for precision was not significant (B = 0.83, SE = 3.05, t = 0.27, *p* = 0.787). The intercept was also non-significant (B = 0.24, SE = 2.03, t = 0.12, *p* = 0.906). Model fit was negligible (R^2^ = 0.001, adjusted R^2^ = −0.010), and the overall F-test was not significant (F(1, 95) = 0.073, *p* = 0.787). These results indicate no evidence of small-study effects or funnel plot asymmetry, suggesting that publication bias is unlikely.

## 4. Discussion

Our systematic review and meta-analysis provide robust evidence that EF among BC survivors is dynamic and influenced by time since surgery, surgical modality, and age. Overall, survivors reported moderately high EF (mean = 73.44), yet lower scores were observed during the early post-surgical period (first 6 months) and at later follow- up (55–72 months). This temporal pattern highlights both the initial adjustment challenges and potential long-term declines in EF, emphasizing the need for extended psychosocial support throughout survivorship. This sets the foundation for understanding how EF changes across time and under different clinical conditions.

In interpreting the changes observed in the EORTC QLQ-C30 scores in the present study, it is important to consider the concept of the minimally important difference (MID) —defined as the smallest change in a score that patients perceive as beneficial (or harmful) and which would lead to a change in patient management [62]. Early guidelines for the QLQ-C30 often adopted a threshold of approximately ten points (on the 0–100 scale) for a clinically meaningful change across all scales [63,64].

More recent work, however, has shown that MIDs for the QLQ-C30 are not uniform: they vary by specific functional or symptom scale, by direction of change (improvement vs. deterioration), by cancer type, and by whether the change is within-group (pre-post) or between-group [62]. For BC populations, estimated MIDs for within-group change generally range from 5 to 14 points for improvement and −4 to −14 points for deterioration across most functional and symptom scales of the QLQ-C30 [65,66]. These thresholds provide valuable context for interpreting whether observed changes in health-related QoL are not only statistically significant but also clinically meaningful from the patient’s perspective.

The longitudinal trajectory analyses revealed a consistent inverted-U pattern, with EF improving during the first 24–30 months post-surgery and gradually declining to near early post-surgical levels by 60 months. This pattern extends previous reports showing significant early psychological adaptation following surgery [21,49] and aligns with broader survivorship literature suggesting that later emotional vulnerability may emerge due to persistent concerns such as long-term treatment effects, fear of recurrence, and body image challenges [2,3,5,6]. Our Quadratic modeling effectively captured these dynamics, underscoring that early postoperative gains in EF may not be sustained in the long term. These findings reinforce calls for continuous emotional monitoring beyond the first two years and transition to a discussion of surgical determinants of EF.

Surgical modality emerged as a key determinant of EF trajectories. Patients who underwent BCS or MA exhibited a characteristic inverted-U pattern, whereas those receiving Mx + IR or other procedures maintained relatively stable EF. These findings suggest that surgical type influences both the magnitude and temporal course of EF recovery, pointing to the value of procedure-specific psychosocial interventions. Consistent with prior findings, patients undergoing BCS generally reported higher EF compared to those undergoing MA, both with and without reconstruction [16,17,20,27]. While Hassan et al. (2024) demonstrated cross-sectional differences in QoL between mastectomy with and without reconstruction—showing slightly better overall QoL and social functioning among women without reconstruction—the study did not assess EF trajectories over time nor include a BCS group, limiting direct comparisons across modalities [22]. Our meta-analysis adds longitudinal context, illustrating how EF evolves differently depending on surgical type.

Specifically, BCS and MA groups exhibited early improvements in EF followed by later stabilization or mild decline, whereas Mx + IR patients demonstrated more stable trajectories over time [21,54]. These findings suggest that the type of surgery shapes both the magnitude and temporal course of emotional recovery, highlighting the importance of procedure-specific psychosocial interventions. Building on these results, age also emerged as a significant moderator of emotional recovery.

Our analysis demonstrated that age moderates EF recovery. Younger (<45 years) and middle-aged (45–60 years) patients experienced early improvements followed by gradual declines, whereas older patients (>60 years) maintained high and stable EF. These findings align with prior studies [18,28,48] showing that younger and middle-aged women experience greater psychosocial distress compared to older patients, largely driven by heightened concerns about body image and social roles. Such distress may also stem from life-stage challenges, including fertility concerns, career disruption, and shifting family or social roles, highlighting the importance of addressing age-specific psychosocial dynamics. Younger and middle-aged survivors, in particular, experience unique emotional vulnerabilities related to role demands, body image, and life-stage transitions, underscoring the need for tailored counseling and survivorship planning.

Our meta-regression further highlighted that middle-aged patients are particularly vulnerable to long-term declines, and that MA is associated with significantly lower EF relative to BCS. This emphasizes the need for age-tailored interventions to optimize recovery of EF. Several studies also underline the interplay between EF and psychosocial factors such as body image, social support, and sexual functioning. For instance, Aerts et al. (2014) reported that women undergoing mastectomy experienced greater challenges in sexual and emotional functioning than those receiving BCS, highlighting the broader psychosocial consequences of surgery type [17]. Similarly, Kouwenberg et al. (2020) and Moro-Valdezate et al. (2014) found that post-surgical complications and cosmetic outcomes significantly impacted emotional well-being, reinforcing the notion that clinical and patient-centered outcomes are closely linked in shaping long-term EF [25,26]. Collectively, these findings underscore the complex, time-dependent nature of EF recovery in BC survivors [7], leading into the broader role of psychosocial resources.

Beyond surgical approaches, patient-reported EF and overall QoL are independently shaped by adjuvant therapies, with both endocrine therapy and chemotherapy producing lasting, domain-specific QoL impairments that persist for years following diagnosis [67,68]. Patients with lower cognitive functioning, adverse life experiences, and limited social support report significantly higher anxiety and depression during radiotherapy, highlighting the pivotal role of psychosocial and cognitive factors in emotional adaptation to treatment [69]. Additionally, disease biology and burden markedly influence these outcomes, with more aggressive breast cancer subtypes consistently linked to poorer QoL and heightened psychosocial distress [70].

Alongside biological and treatment-related influences, psychosocial factors play a pivotal role in shaping EF trajectories among BC survivors [3,41,71,72]. Studies have consistently demonstrated that emotional support, coping strategies, and social networks significantly influence psychological outcomes. For instance, Devarakonda et al. (2023) found that EF improved over time, particularly among survivors who were engaged in strong social support networks and utilized adaptive coping mechanisms, whereas limited support and maladaptive coping were linked to persistent distress [3]. Similarly, Zamanian et al. (2021) demonstrated that higher perceived social support was associated with lower anxiety and depression in women with BC, and that adaptive coping mediated this relationship, highlighting the critical role of psychosocial resources in supporting long-term EF [71]. These insights point toward the therapeutic potential of structured psychosocial interventions.

Indeed, evidence underscores the importance of structured psychosocial interventions, such as cognitive–behavioral therapy and supportive counseling, in improving emotional outcomes among cancer survivors, particularly when implemented early in survivorship [72]. Moreover, fostering strong social networks and promoting adaptive coping skills may buffer against negative emotional impacts of treatment-related stressors, body image concerns, and fear of recurrence. Personalized approaches that consider age, surgical modality, social support, and life-stage-specific challenges are likely to be most effective in promoting sustained emotional recovery. Implementing such tailored psychosocial interventions within multidisciplinary cancer care may optimize long-term QoL and enhance overall survivorship outcomes.

### 4.1. Strengths and Limitations

This meta-analysis modeled long-term trajectories of EF in BC survivors across surgical modalities and age groups using multilevel random-effects models. Its robustness is supported by adherence to PRISMA guidelines, use of standardized EF measures (EORTC QLQ-C30). Nevertheless, heterogeneity in follow-up durations and reliance on a single EF instrument may have influenced pooled estimates. In addition, the use of aggregate-level data limited adjustment for key confounders such as adjuvant therapy and baseline psychological status, suggesting directions for methodological refinement in future research.

### 4.2. Future Directions

Future studies should clarify the mechanisms underlying the inverted-U trajectory of EF, focusing on factors contributing to late-phase declines. Biopsychosocial variables—such as fatigue, endocrine symptoms, fear of recurrence, and limited social support—likely interact over time to shape emotional adaptation. Given the increased vulnerability observed among middle-aged women, further research should address age-specific stressors, including caregiving demands, occupational strain, and menopausal transitions. Qualitative and mixed-methods approaches are recommended to capture nuanced emotional experiences and inform the design of targeted, age-sensitive psychosocial interventions, bridging research and practical survivorship care.

While psychosocial determinants play a critical role in EF outcomes, biomedical and treatment-related factors also contribute substantially. To capture the full complexity of these trajectories, future research should adopt an integrative approach that considers the cumulative and interactive effects of adjuvant therapies (e.g., chemotherapy, radiotherapy, and endocrine therapy), tumor characteristics, and clinical prognostic factors (such as lymph node status and recurrence risk) on EF. Examining these variables in conjunction with surgical and psychosocial factors will be critical to elucidating the multidimensional pathways influencing recovery and long-term well-being among BC survivors.

## 5. Conclusions

This study demonstrates that EF among BC survivors is dynamic and shaped by time since surgery, surgical modality, and age. Overall EF was moderately high but followed an inverted-U trajectory, with early post-surgical improvement followed by later decline. Patients undergoing BCS or MA showed similar curvilinear patterns, while those receiving immediate reconstruction maintained stable EF. Younger and middle-aged survivors exhibited greater fluctuations compared with older patients, underscoring their vulnerability to long-term emotional decline. These findings highlight the importance of sustained, age- and surgery-specific psychosocial support beyond the early recovery phase to promote enduring emotional well-being and optimize QoL throughout survivorship.

## Figures and Tables

**Table 1:** Multivariate Meta-Regression Model of Emotional Functioning Trajectories by Age Group and Surgical Modality.

Predictor	Estimate ± SE	z-Value	*p*-Value	95% CI
Intercept	76.42 ± 2.97	25.77	<0.001	[70.60, 82.23]
Time (linear)	11.51 ± 3.24	3.55	0.0004	[5.16, 17.85]
Time^2^ (quadratic)	−5.37 ± 2.15	−2.50	0.013	[−9.58, −1.16]
Age >60 years	0.74 ± 2.30	0.32	0.748	[−3.76, 5.24]
Age 45–60 years	2.30 ± 1.44	1.60	0.109	[−0.51, 5.12]
Age Unknown	7.28 ± 2.74	2.66	0.008	[1.91, 12.65]
Mastectomy Alone	−1.87 ± 0.81	−2.30	0.022	[−3.46, −0.27]
Mastectomy IBR	−2.97 ± 1.89	−1.57	0.117	[−6.68, 0.74]
Other Surgery	−6.24 ± 4.74	−1.32	0.188	[−15.53, 3.06]
Time × Age 45–60 years	−4.64 ± 0.94	−4.92	<0.001	[−6.48, −2.79]
Time × Age Unknown	−4.15 ± 2.92	−1.42	0.156	[−9.87, 1.58]
Time^2^ × Mastectomy Alone	−0.13 ± 0.78	−0.17	0.865	[−1.65, 1.39]
Time^2^ × Mastectomy IBR	0.97 ± 1.48	0.65	0.514	[−1.94, 3.87]
Time^2^ × Other Surgery	6.02 ± 3.54	1.70	0.089	[−0.91, 12.95]
Time × Mastectomy Alone	0.67 ± 1.13	0.59	0.555	[−1.55, 2.89]
Time × Mastectomy IBR	−0.95 ± 2.53	−0.37	0.708	[−5.90, 4.01]
Time × Other Surgery	−9.65 ± 5.48	−1.76	0.079	[−20.40, 1.10]

Footnote: Reference categories were patients <45 years and breast-conserving surgery. Time was modeled with standardized linear and quadratic terms. All estimates reflect differences relative to these baseline groups.

## Data Availability

Data supporting the results of this study are available from the corresponding author upon reasonable request.

## Supplementary Materials

The following supporting information can be downloaded at: https://www.mdpi.com/article/10.3390/cancers17223707/s1, Figure S1: PRISMA flow diagram; Table S1: Individual Effect Measures [12]; Table S2: Studies Internal Validity; Table S3: Search strategy string.

## Abbreviations

The following abbreviations are used in this manuscript:

AIC	Akaike information criterion
BC	breast cancer
BCS	breast-conserving surgery
BIC	Bayesian information criterion
EF	emotional functioning
IBR	immediate breast reconstruction
MA	mastectomy alone
MID	minimally important difference
Mx + IR	mastectomy with immediate reconstruction
QoL	quality of life

## Appendix A

**Table A1:** Subgroup Analysis of EF by Time Points, Surgery Type, and Age Group.

Group	k	Studies	Estimate ± SE	95% CI	I^2^ (%)	*p*-Value
Overall	116	40	73.44 ± 1.61	[70.29, 76.58]	0.0	<0.001
Time since surgery						
0–6 months	34	13	66.82 ± 3.61	[59.75, 73.89]	93.2	<0.001
7–15 months	34	16	77.86 ± 1.71	[74.51, 81.22]	80.2	<0.001
16–30 months	10	5	72.58 ± 5.68	[61.45, 83.72]	91.0	<0.001
31–54 months	10	6	77.52 ± 3.61	[70.44, 84.59]	81.5	<0.001
55–72 months	9	4	69.81 ± 2.92	[64.08, 75.54]	76.6	<0.001
Unknown	19	9	74.82 ± 5.42	[64.20, 85.43]	95.9	<0.001
Surgical modality						
Breast-conserving	41	28	74.40 ± 2.01	[70.45, 78.34]	23.1	<0.001
Mastectomy alone	45	29	71.93 ± 2.23	[67.57, 76.30]	0.0	<0.001
Mastectomy IBR	26	13	72.17 ± 2.93	[66.42, 77.92]	0.0	<0.001
Other	4	4	69.92 ± 2.26	[65.48, 74.36]	56.8	<0.001
Age group						
45–60 years	79	32	70.68 ± 1.81	[67.14, 74.22]	0.0	<0.001
<45 years	11	6	74.85 ± 2.79	[69.37, 80.32]	83.1	<0.001
>60 years	8	6	79.00 ± 3.49	[72.15, 85.84]	87.0	<0.001
Unknown	18	6	81.66 ± 2.23	[77.28, 86.03]	0.0	<0.001

Footnote: Mean EF estimates are based on pooled scores using a 0–100 harmonized scale, where higher values indicate better EF. Random-effects multilevel meta-analyses were conducted using REML estimation. I^2^ represents the proportion of total variability due to between-study heterogeneity. All *p*-values refer to the test of pooled effects within each subgroup.

**Table A2:** Overall trajectory modeling results for EF.

Model	AIC	BIC	ΔAIC	ΔBIC
Quadratic	10977.48	10994.90	0.00	0.00
Logarithmic	10991.38	11005.34	13.90	10.44
Linear	10997.74	11011.70	20.26	16.80

Footnote: AIC = Akaike Information Criterion; BIC = Bayesian Information Criterion. Lower values indicate better model fit. ΔAIC and ΔBIC reflect differences relative to the best-fitting model (quadratic). R^2^ reflects explained variance.

**Table A3:** Trajectory Model Estimates for EF by Surgical Modality.

Surgery Type	k	Studies	Intercept ± SE	Linear Term ± SE	Quadratic Term ± SE	QM *p*-Value	τ^2^	QE (*p*-Value)
Breast-Conserving	41	28	63.64 ± 4.81 ***	1.22 ± 0.50 *	−0.02 ± 0.01 *	0.046	0.00	1253.34 (<0.001)
Mastectomy Alone	45	29	61.29 ± 4.64 ***	1.19 ± 0.51 *	−0.02 ± 0.01 *	0.054	0.00	3688.77 (<0.001)
Mastectomy IBR	26	13	71.46 ± 4.46 ***	0.11 ± 0.20	—	0.582	0.00	481.60 (<0.001)
Other	4	4	66.10 ± 1.86 ***	0.07 ± 0.03	—	0.053	0.00	0.97 (0.325)

Footnote: All models were unadjusted and fitted independently within each surgery subgroup using quadratic or linear terms depending on fit. EF estimates are on a 0–100 scale. QM *p*-values refer to model significance. QE tests for unexplained variance. Significance codes: *** *p* < 0.001, * *p* < 0.05.

**Table A4:** Trajectory Model Estimates for EF by Age Group.

Age Group	k	Studies	Intercept ± SE	Linear Term ± SE	Quadratic Term ± SE	QM *p*-Value	τ^2^	QE (*p*-Value)
45–60 years	79	32	63.34 ± 3.55 ***	0.87 ± 0.38 *	–0.01 ± 0.01 *	0.067	0.00	3339.14 (<0.001)
<45 years	11	6	67.56 ± 4.26 ***	0.82 ± 0.34 *	–0.01 ± 0.01 *	0.051	0.00	361.06 (<0.001)
>60 years	8	6	75.60 ± 5.18 ***	—	—	<0.001	0.00	50.45 (<0.001)
Unknown	18	6	72.27 ± 4.92 ***	1.32 ± 0.58 *	–0.02 ± 0.01 *	0.075	0.00	138.24 (<0.001)

Footnote: Models were independently fitted within each age group. All estimates are based on a 0–100 scale. QM *p*-values reflect model-level significance. QE tests for within-group variance unexplained by time. Significance codes: *** *p* < 0.001, * *p* < 0.05.

**Table A5:** Omnibus Test of Moderators for EF.

Moderator	Chi-Square	df	*p*-Value
Time Points	13.419	4	0.009
Surgery Types	11.264	3	0.010
Age Groups	15.318	3	0.002
Study Design	2.818	4	0.589

Footnote: Tests conducted using mixed-effects meta-regression models. Chi-square statistics assess whether each moderator explains a significant proportion of between-study heterogeneity. Statistical significance was set at *p* < 0.05.
