# Supplementary material for: Emotional Functioning as a Dimension of Quality of Life in Breast Cancer Survivors: A Systematic Review and Meta-Analysis
Source: Cancers (Basel). 2025 Nov 19;17(22):3707. doi: 10.3390/cancers17223707 (PMC12650911; doi:10.3390/cancers17223707)
Supplement: Supplementary file 1 [file cancers-17-03707-s001.zip › Figure S1. Prisma Flow Chart.pdf]

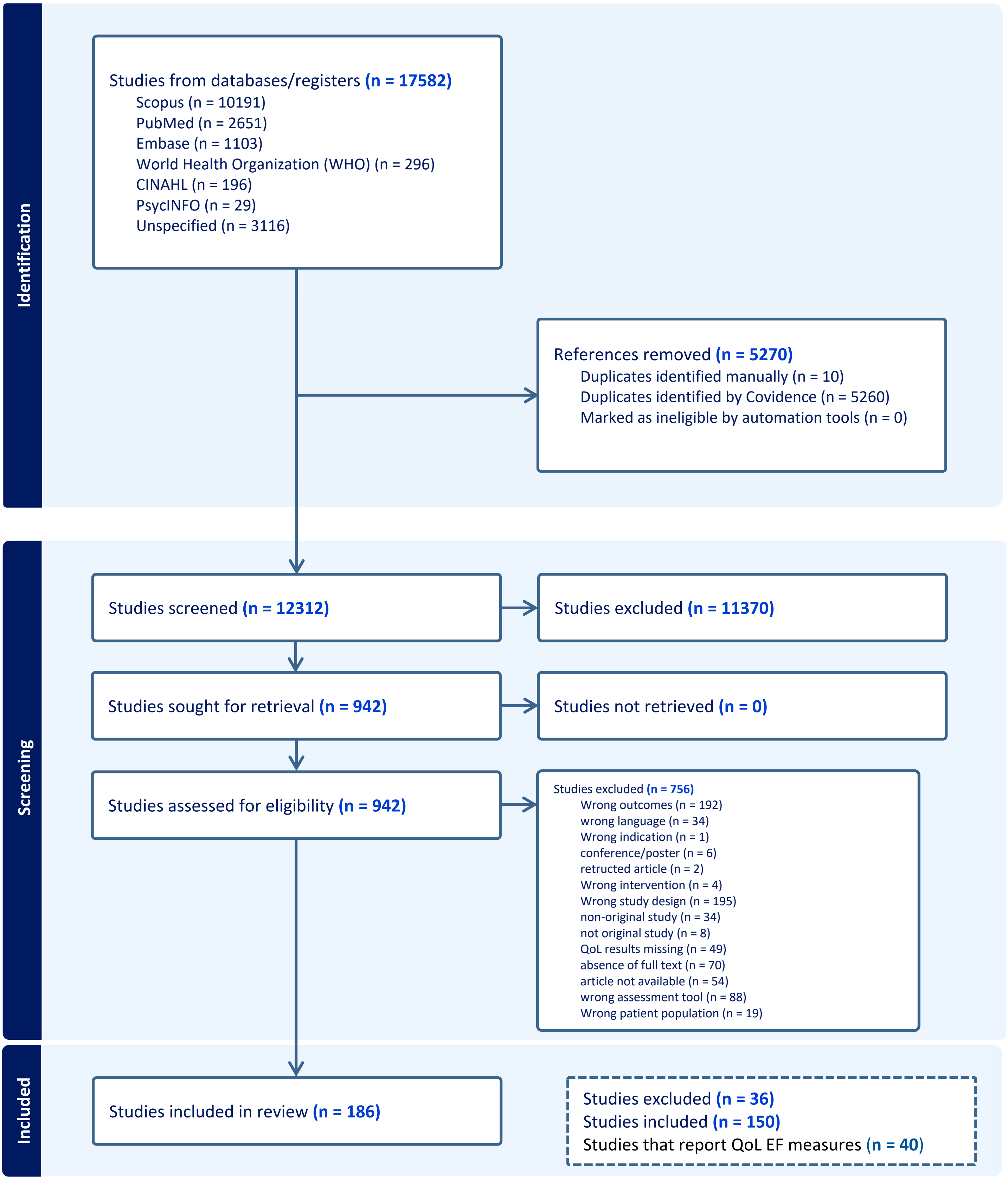

Figure S1. Prisma Flow Chart.

covidence
