# Supplementary material for: Emotional Functioning as a Dimension of Quality of Life in Breast Cancer Survivors: A Systematic Review and Meta-Analysis
Source: Cancers (Basel). 2025 Nov 19;17(22):3707. doi: 10.3390/cancers17223707 (PMC12650911; doi:10.3390/cancers17223707)
Supplement: Supplementary file 1 [file cancers-17-03707-s001.zip › Table S2. Studies Internal Validity.pdf]

Table S2. Studies Internal Validity.

Internal Validity Appraisal JBI

Acil, 2014

Criteria	Yes	No	Unclear
1. Were the criteria for inclusion in the sample clearly defined?	*		
2. Were the study subjects and the setting described in detail?	*		
3. Was the exposure measured in a valid and reliable way?	*		
4. Were objective, standard criteria used for measurement of the condition?	*		
5. Were confounding factors identified?	*		
6. Were strategies to deal with confounding factors stated?	*		
7. Were the outcomes measured in a valid and reliable way?	*		
8. Was appropriate statistical analysis used?	*		

Aerts, 2014

Criteria	Yes	No	Unclear
1. Were the criteria for inclusion in the sample clearly defined?	*		
2. Were the study subjects and the setting described in detail?	*		
3. Was the exposure measured in a valid and reliable way?	*		
4. Were objective, standard criteria used for measurement of the condition?	*		
5. Were confounding factors identified?	*		
6. Were strategies to deal with confounding factors stated?	*		
7. Were the outcomes measured in a valid and reliable way?	*		
8. Was appropriate statistical analysis used?	*		

Cherian, 2022

Criteria	Yes	No	Unclear
1. Were the criteria for inclusion in the sample clearly defined?	*		
2. Were the study subjects and the setting described in detail?	*		
3. Was the exposure measured in a valid and reliable way?	*		
4. Were objective, standard criteria used for measurement of the condition?	*		
5. Were confounding factors identified?	*		
6. Were strategies to deal with confounding factors stated?	*		
7. Were the outcomes measured in a valid and reliable way?	*		
8. Was appropriate statistical analysis used?	*		

Cortés-Flores, 2014

Criteria	Yes	No	Unclear
1. Were the criteria for inclusion in the sample clearly defined?	*		
2. Were the study subjects and the setting described in detail?	*		
3. Was the exposure (type of surgery) measured in a valid and reliable way?	*		
4. Were objective, standard criteria used for measurement of the condition (quality of life)?	*		
5. Were confounding factors identified?	*		
6. Were strategies to deal with confounding factors stated?			*
7. Were the outcomes measured in a valid and reliable way?	*		
8. Was appropriate statistical analysis used?	*		

Dahlui, 2023

Criteria	Yes	No	Unclear
1. Were the criteria for inclusion in the sample clearly defined?	*		
2. Were the study subjects and the setting described in detail?	*		
3. Was the exposure (type of surgery) measured in a valid and reliable way?	*		
4. Were objective, standard criteria used for measurement of the condition (quality of life)?	*		
5. Were confounding factors identified?	*		
6. Were strategies to deal with confounding factors stated?	*		
7. Were the outcomes measured in a valid and reliable way?	*		
8. Was appropriate statistical analysis used?	*		

Harcourt, 2003

Criteria	Yes	No	Unclear
1. Were the criteria for inclusion in the sample clearly defined?	*		
2. Were the study subjects and the setting described in detail?	*		
3. Was the exposure (mastectomy with or without reconstruction) measured in a valid and reliable way?	*		
4. Were objective, standard criteria used for measurement of the condition (psychological outcomes)?	*		
5. Were confounding factors identified?	*		
6. Were strategies to deal with confounding factors stated?			*
7. Were the outcomes measured in a valid and reliable way?	*		
8. Was appropriate statistical analysis used?	*		

Nowicki, 2015

Criteria	Yes	No	Unclear
1. Were the criteria for inclusion in the sample clearly defined?	*		
2. Were the study subjects and the setting described in detail?	*		
3. Was the exposure (type of surgery: mastectomy vs. breast conserving surgery) measured in a valid and reliable way?	*		
4. Were objective, standard criteria used for measurement of the condition (QoL)?	*		
5. Were confounding factors identified?			*
6. Were strategies to deal with confounding factors stated?			*
7. Were the outcomes measured in a valid and reliable way?	*		
8. Was appropriate statistical analysis used?	*		

Songtish, 2021.

Question	Yes	No	Unclear
1. Were the criteria for inclusion in the sample clearly defined?	*		
2. Were the study subjects and the setting described in detail?	*		
3. Was the exposure measured in a valid and reliable way?	*		
4. Were objective, standard criteria used for measurement of the condition?	*		
5. Were confounding factors identified?			*
6. Were strategies to deal with confounding factors stated?			*
7. Were the outcomes measured in a valid and reliable way?	*		
8. Was appropriate statistical analysis used?	*		

Cohen, 2000

Question	Yes	No	Unclear
1. Were the criteria for inclusion in the sample clearly defined?	*		
2. Were the study subjects and the setting described in detail?	*		
3. Was the exposure measured in a valid and reliable way?	*		
4. Were objective, standard criteria used for measurement of the condition?	*		
5. Were confounding factors identified?	*		
6. Were strategies to deal with confounding factors stated?	*		
7. Were the outcomes measured in a valid and reliable way?	*		
8. Was appropriate statistical analysis used?	*		

Domenici, 2022

Question	Yes	No	Unclear
1. Were the criteria for inclusion in the sample clearly defined?	*		
2. Were the study subjects and the setting described in detail?	*		
3. Was the exposure measured in a valid and reliable way?	*		
4. Were objective, standard criteria used for measurement of the condition?	*		
5. Were confounding factors identified?			*
6. Were strategies to deal with confounding factors stated?			*
7. Were the outcomes measured in a valid and reliable way?	*		
8. Was appropriate statistical analysis used?	*		

Hejl, 2021

Question	Yes	No	Unclear
1. Were the criteria for inclusion in the sample clearly defined?	*		
2. Were the study subjects and the setting described in detail?	*		
3. Was the exposure measured in a valid and reliable way?	*		
4. Were objective, standard criteria used for measurement of the condition?	*		
5. Were confounding factors identified?	*		
6. Were strategies to deal with confounding factors stated?			*
7. Were the outcomes measured in a valid and reliable way?	*		
8. Was appropriate statistical analysis used?	*		

Hadi, 2012

Question	Yes	No	Unclear
1. Were the criteria for inclusion in the sample clearly defined?	*		
2. Were the study subjects and the setting described in detail?	*		
3. Was the exposure measured in a valid and reliable way?	*		
4. Were objective, standard criteria used for measurement of the condition?	*		
5. Were confounding factors identified?			*
6. Were strategies to deal with confounding factors stated?			*
7. Were the outcomes measured in a valid and reliable way?	*		
8. Was appropriate statistical analysis used?	*		

Hassan, 2024

Question	Yes	No	Unclear
1. Were the criteria for inclusion in the sample clearly defined?	*		
2. Were the study subjects and the setting described in detail?	*		
3. Was the exposure measured in a valid and reliable way?	*		
4. Were objective, standard criteria used for measurement of the condition?	*		
5. Were confounding factors identified?			*
6. Were strategies to deal with confounding factors stated?			*
7. Were the outcomes measured in a valid and reliable way?	*		
8. Was appropriate statistical analysis used?	*		

Jayasinghe, 2021

Question	Yes	No	Unclear
1. Were the criteria for inclusion in the sample clearly defined?	*		
2. Were the study subjects and the setting described in detail?	*		
3. Was the exposure measured in a valid and reliable way?	*		
4. Were objective, standard criteria used for measurement of the condition?	*		
5. Were confounding factors identified?			*
6. Were strategies to deal with confounding factors stated?			*
7. Were the outcomes measured in a valid and reliable way?	*		
8. Was appropriate statistical analysis used?	*		

Konieczny, 2023

Question	Yes	No	Unclear
1. Were the criteria for inclusion in the sample clearly defined?	*		
2. Were the study subjects and the setting described in detail?	*		
3. Was the exposure measured in a valid and reliable way?	*		
4. Were objective, standard criteria used for measurement of the condition?	*		
5. Were confounding factors identified?			*
6. Were strategies to deal with confounding factors stated?			*
7. Were the outcomes measured in a valid and reliable way?	*		
8. Was appropriate statistical analysis used?	*		

Kouwenberg, 2020

Question	Yes	No	Unclear
1. Were the criteria for inclusion in the sample clearly defined?	*		
2. Were the study subjects and the setting described in detail?	*		
3. Was the exposure measured in a valid and reliable way?	*		
4. Were objective, standard criteria used for measurement of the condition?	*		
5. Were confounding factors identified?	*		
6. Were strategies to deal with confounding factors stated?	*		
7. Were the outcomes measured in a valid and reliable way?	*		
8. Was appropriate statistical analysis used?	*		

Moro-Valdezate, 2012

Question	Yes	No	Unclear
1. Were the criteria for inclusion in the sample clearly defined?	*		
2. Were the study subjects and the setting described in detail?	*		
3. Was the exposure measured in a valid and reliable way?	*		
4. Were objective, standard criteria used for measurement of the condition?	*		
5. Were confounding factors identified?	*		
6. Were strategies to deal with confounding factors stated?	*		
7. Were the outcomes measured in a valid and reliable way?	*		
8. Was appropriate statistical analysis used?	*		

Nsaful, 2024

Question	Yes	No	Unclear
1. Were the criteria for inclusion in the sample clearly defined?	*		
2. Were the study subjects and the setting described in detail?	*		
3. Was the exposure measured in a valid and reliable way?	*		
4. Were objective, standard criteria used for measurement of the condition?	*		
5. Were confounding factors identified?	*		
6. Were strategies to deal with confounding factors stated?	*		
7. Were the outcomes measured in a valid and reliable way?	*		
8. Was appropriate statistical analysis used?	*		

Pačarić, 2018

Question	Yes	No	Unclear
1. Were the criteria for inclusion in the sample clearly defined?	*		
2. Were the study subjects and the setting described in detail?	*		
3. Was the exposure measured in a valid and reliable way?	*		
4. Were objective, standard criteria used for measurement of the condition?	*		
5. Were confounding factors identified?	*		
6. Were strategies to deal with confounding factors stated?	*		
7. Were the outcomes measured in a valid and reliable way?	*		
8. Was appropriate statistical analysis used?	*		

Camejo, 2024

Question	Yes	No	Unclear
1. Were the criteria for inclusion in the sample clearly defined?	*		
2. Were the study subjects and the setting described in detail?	*		
3. Was the exposure measured in a valid and reliable way?	*		
4. Were objective, standard criteria used for measurement of the condition?	*		
5. Were confounding factors identified?	*		
6. Were strategies to deal with confounding factors stated?	*		
7. Were the outcomes measured in a valid and reliable way?	*		
8. Was appropriate statistical analysis used?	*		

Enien, 2018

Question	Yes	No	Unclear
1. Were the criteria for inclusion in the sample clearly defined?	*		
2. Were the study subjects and the setting described in detail?	*		
3. Was the exposure measured in a valid and reliable way?	*		
4. Were objective, standard criteria used for measurement of the condition?	*		
5. Were confounding factors identified?	*		
6. Were strategies to deal with confounding factors stated?	*		
7. Were the outcomes measured in a valid and reliable way?	*		
8. Was appropriate statistical analysis used?	*		

Esgueva, 2022

Question	Yes	No	Unclear
1. Were the criteria for inclusion in the sample clearly defined?	*		
2. Were the study subjects and the setting described in detail?	*		
3. Was the exposure measured in a valid and reliable way?	*		
4. Were objective, standard criteria used for measurement of the condition?	*		
5. Were confounding factors identified?	*		
6. Were strategies to deal with confounding factors stated?	*		
7. Were the outcomes measured in a valid and reliable way?	*		
8. Was appropriate statistical analysis used?	*		

Gillies, 2023

Question	Yes	No	Unclear
1. Were the criteria for inclusion in the sample clearly defined?	*		
2. Were the study subjects and the setting described in detail?	*		
3. Was the exposure measured in a valid and reliable way?	*		
4. Were objective, standard criteria used for measurement of the condition?	*		
5. Were confounding factors identified?	*		
6. Were strategies to deal with confounding factors stated?	*		
7. Were the outcomes measured in a valid and reliable way?	*		
8. Was appropriate statistical analysis used?	*		

Lagendijk, 2018

Question	Yes	No	Unclear
1. Were the criteria for inclusion in the sample clearly defined?	*		
2. Were the study subjects and the setting described in detail?	*		
3. Was the exposure measured in a valid and reliable way?	*		
4. Were objective, standard criteria used for measurement of the condition?	*		
5. Were confounding factors identified?	*		
6. Were strategies to deal with confounding factors stated?	*		
7. Were the outcomes measured in a valid and reliable way?	*		
8. Was appropriate statistical analysis used?	*		

Kim, 2015

Question	Yes	No	Unclear
1. Were the criteria for inclusion in the sample clearly defined?	*		
2. Were the study subjects and the setting described in detail?	*		
3. Was the exposure measured in a valid and reliable way?	*		
4. Were objective, standard criteria used for measurement of the condition?	*		
5. Were confounding factors identified?	*		
6. Were strategies to deal with confounding factors stated?	*		
7. Were the outcomes measured in a valid and reliable way?	*		
8. Was appropriate statistical analysis used?	*		

Qin, 2018

Question	Yes	No	Unclear
1. Were the criteria for inclusion in the sample clearly defined?	*		
2. Were the study subjects and the setting described in detail?	*		
3. Was the exposure measured in a valid and reliable way?	*		
4. Were objective, standard criteria used for measurement of the condition?	*		
5. Were confounding factors identified?	*		
6. Were strategies to deal with confounding factors stated?	*		
7. Were the outcomes measured in a valid and reliable way?	*		
8. Was appropriate statistical analysis used?	*		

Shi, 2011

Question	Yes	No	Unclear
1. Were the criteria for inclusion in the sample clearly defined?	*		
2. Were the study subjects and the setting described in detail?	*		
3. Was the exposure measured in a valid and reliable way?	*		
4. Were objective, standard criteria used for measurement of the condition?	*		
5. Were confounding factors identified?	*		
6. Were strategies to deal with confounding factors stated?	*		
7. Were the outcomes measured in a valid and reliable way?	*		
8. Was appropriate statistical analysis used?	*		

Sun, 2014

Question	Yes	No	Unclear
1. Were the criteria for inclusion in the sample clearly defined?	*		
2. Were the study subjects and the setting described in detail?	*		
3. Was the exposure measured in a valid and reliable way?	*		
4. Were objective, standard criteria used for measurement of the condition?	*		
5. Were confounding factors identified?	*		
6. Were strategies to deal with confounding factors stated?	*		
7. Were the outcomes measured in a valid and reliable way?	*		

Question	Yes	No	Unclear
8. Was appropriate statistical analysis used?	*		

Ozmen, 2020

Question	Yes	No	Unclear
1. Were the criteria for inclusion in the sample clearly defined?	*		
2. Were the study subjects and the setting described in detail?	*		
3. Was the exposure measured in a valid and reliable way?	*		
4. Were objective, standard criteria used for measurement of the condition?	*		
5. Were confounding factors identified?	*		
6. Were strategies to deal with confounding factors stated?	*		
7. Were the outcomes measured in a valid and reliable way?	*		
8. Was appropriate statistical analysis used?	*		

Spatuzzi, 2016

Question	Yes	No	Unclear
1. Were the criteria for inclusion in the sample clearly defined?	*		
2. Were the study subjects and the setting described in detail?	*		
3. Was the exposure measured in a valid and reliable way?	*		
4. Were objective, standard criteria used for measurement of the condition?	*		
5. Were confounding factors identified?		*	
6. Were strategies to deal with confounding factors stated?		*	
7. Were the outcomes measured in a valid and reliable way?	*		
8. Was appropriate statistical analysis used?	*		

Tsai, 2017

Question	Yes	No	Unclear
1. Were the criteria for inclusion in the sample clearly defined?	*		
2. Were the study subjects and the setting described in detail?	*		
3. Was the exposure measured in a valid and reliable way?	*		
4. Were objective, standard criteria used for measurement of the condition?	*		
5. Were confounding factors identified?		*	
6. Were strategies to deal with confounding factors stated?		*	

Question	Yes	No	Unclear
7. Were the outcomes measured in a valid and reliable way?	*		
8. Was appropriate statistical analysis used?	*		

Razdan, 2024

Question	Yes	No	Unclear
1. Were the criteria for inclusion in the sample clearly defined?	*		
2. Were the study subjects and the setting described in detail?	*		
3. Was the exposure measured in a valid and reliable way?	*		
4. Were objective, standard criteria used for measurement of the condition?	*		
5. Were confounding factors identified?		*	
6. Were strategies to deal with confounding factors stated?		*	
7. Were the outcomes measured in a valid and reliable way?	*		
8. Was appropriate statistical analysis used?	*		

Volders, 2017

Question	Yes	No	Unclear
1. Were the criteria for inclusion in the sample clearly defined?	*		
2. Were the study subjects and the setting described in detail?	*		
3. Was the exposure measured in a valid and reliable way?	*		
4. Were objective, standard criteria used for measurement of the condition?	*		
5. Were confounding factors identified?		*	
6. Were strategies to deal with confounding factors stated?		*	
7. Were the outcomes measured in a valid and reliable way?	*		
8. Was appropriate statistical analysis used?	*		

von Glinski, 2022

Question	Yes	No	Unclear
1. Were the criteria for inclusion in the sample clearly defined?	*		
2. Were the study subjects and the setting described in detail?	*		
3. Was the exposure measured in a valid and reliable way?	*		
4. Were objective, standard criteria used for measurement of the condition?	*		
5. Were confounding factors identified?		*	
6. Were strategies to deal with confounding factors stated?		*	
7. Were the outcomes measured in a valid and reliable way?	*		
8. Was appropriate statistical analysis used?	*		

Janni, 2001

Question	Yes	No	Unclear
1. Were the criteria for inclusion in the sample clearly defined?	*		
2. Were the study subjects and the setting described in detail?	*		
3. Was the exposure measured in a valid and reliable way?	*		
4. Were objective, standard criteria used for measurement of the condition?	*		
5. Were confounding factors identified?	*		
6. Were strategies to deal with confounding factors stated?	*		
7. Were the outcomes measured in a valid and reliable way?	*		
8. Was appropriate statistical analysis used?	*		

King, 2000

Question	Yes	No	Unclear
1. Were the criteria for inclusion in the sample clearly defined?	*		
2. Were the study subjects and the setting described in detail?	*		
3. Was the exposure measured in a valid and reliable way?	*		
4. Were objective, standard criteria used for measurement of the condition?	*		
5. Were confounding factors identified?	*		
6. Were strategies to deal with confounding factors stated?		*	
7. Were the outcomes measured in a valid and reliable way?	*		
8. Was appropriate statistical analysis used?	*		

Szutowicz-Wydra, 2016

Question	Yes	No	Unclear
1. Were the criteria for inclusion in the sample clearly defined?	*		
2. Were the study subjects and the setting described in detail?	*		
3. Was the exposure measured in a valid and reliable way?	*		
4. Were objective, standard criteria used for measurement of the condition?	*		
5. Were confounding factors identified?		*	
6. Were strategies to deal with confounding factors stated?		*	
7. Were the outcomes measured in a valid and reliable way?	*		
8. Was appropriate statistical analysis used?	*		

Rahman, 2015

No.	Appraisal Question	Yes	No	Unclear	Not Applicable
1	Were the criteria for inclusion in the sample clearly defined?	*			
2	Were the study subjects and the setting described in detail?	*			
3	Was the exposure measured in a valid and reliable way?	*			
4	Were objective, standard criteria used for measurement of the condition?	*			
5	Were confounding factors identified?	*			
6	Were strategies to deal with confounding factors stated?			*	
7	Were the outcomes measured in a valid and reliable way?	*			
8	Was appropriate statistical analysis used?			*	

Senoga, 2023

No.	Appraisal Question	Yes	No	Unclear	Not Applicable
1	Were the criteria for inclusion in the sample clearly defined?	*			
2	Were the study subjects and the setting described in detail?	*			
3	Was the exposure measured in a valid and reliable way?	*			
4	Were objective, standard criteria used for measurement of the condition?	*			
5	Were confounding factors identified?	*			
6	Were strategies to deal with confounding factors stated?	*			
7	Were the outcomes measured in a valid and reliable way?	*			
8	Was appropriate statistical analysis used?			*	

Han, 2010

No.	Appraisal Question	Yes	No	Unclear	Not Applicable
1	Were the criteria for inclusion in the sample clearly defined?	*			
2	Were the study subjects and the setting described in detail?	*			
3	Was the exposure measured in a valid and reliable way?	*			
4	Were objective, standard criteria used for measurement of the condition?	*			
5	Were confounding factors identified?			*	
6	Were strategies to deal with confounding factors stated?			*	
7	Were the outcomes measured in a valid and reliable way?	*			
8	Was appropriate statistical analysis used?			*	
