# Supplementary material for: Emotional Functioning as a Dimension of Quality of Life in Breast Cancer Survivors: A Systematic Review and Meta-Analysis
Source: Cancers (Basel). 2025 Nov 19;17(22):3707. doi: 10.3390/cancers17223707 (PMC12650911; doi:10.3390/cancers17223707)
Supplement: Supplementary file 1 [file cancers-17-03707-s001.zip › Table S3. Search strategy string.pdf]

Table S3. Search strategy string.

Search strategy for PubMed: 04.12.2024

**Search no**	**String**	**Results**	**Notes**
1	((cancer survivors[MeSH Terms]) OR (cancer patients[Title/Abstract])) OR (breast cancer patients[Title/Abstract])	254,719	
2	(((((breast-conserving surgery[Title/Abstract]) OR (breast conservation[Title/Abstract])) OR (breast conserving surgery[Title/Abstract])) OR (lumpectomy[Title/Abstract])) OR (lumpectomy[MeSH Terms])) OR (mastectomy, partial[MeSH Terms])	17,743	
3	(mastectomy[Title/Abstract]) OR (mastectomy[MeSH Terms])	49,938	
4	((quality of life[MeSH Terms]) OR (quality of life[Title/Abstract])) OR (well-being[Title/Abstract])	594,882	
5	#1 AND (#2 OR #3) AND #4	2651	English; 2000 - 2025

Search strategy for Scopus: 04.12.2024

**Search no**	**String**	**Results**	**Notes**
1	(TITLE-ABS-KEY (cancer AND survivors) OR TITLE-ABS-KEY (cancer AND patients) OR TITLE-ABS-KEY (breast AND cancer AND patients))	2,029,003	
2		34,754	

	(TITLE-ABS-KEY (breast-conserving AND surgery) OR TITLE-ABS-KEY (breast AND conservation) OR TITLE-ABS-KEY (breast AND conserving AND surgery) OR TITLE-ABS-KEY (lumpectomy) OR TITLE-ABS-KEY (mastectomy, AND partial))		
3	TITLE-ABS-KEY (mastectomy)	77,842	
4	((quality of life[MeSH Terms]) OR (quality of life[Title/Abstract])) OR (well-being[Title/Abstract])	11,887,744	
5	#1 AND (#2 OR #3) AND #4	10,192	English; 2000 – 2025; limit to subject areas: medicine; biochemistry, genetics and molecular biology; nursing; phramacology, toxicology and pharmaceutics; psychology; health professions.

Search strategy for CINAHL, Health Sciences Databases: 04.12.2024

**Search no**	**String**	**Results**	**Notes**
1	TI cancer survivors OR AB cancer survivors OR TI cancer patients OR AB cancer patients OR TI breast cancer patients OR AB breast cancer patients	84,774	
2	TI breast-conserving surgery OR AB breast-conserving surgery OR TI breast conservation OR AB breast conservation OR TI breast conserving surgery OR AB breast conserving surgery OR TI lumpectomy OR AB lumpectomy OR TI mastectomy, partial OR AB mastectomy, partial	3,433	
3	TI mastectomy OR AB mastectomy	6,109	

4	((quality of life[MeSH Terms]) OR (quality of life[Title/Abstract])) OR (well-being[Title/Abstract])	221,539	
5	#2 OR #3	8,311	
6	#1 AND #4 AND #5	196	English, 2000 – 2025.

Search strategy for EMBASE: 04.12.2024

**Search no**	**String**	**Results**	**Notes**
1	**'cancer survivor'**:ab,ti OR **'cancer survivor'**/exp/mj OR **'cancer patients'**:ab,ti OR **'cancer patients'** OR **'breast cancer patients'**:ab,ti OR **'breast cancer patients'**	423,341	
2	**'breast-conserving surgery'**:ab,ti OR **'breast-conserving surgery'**/exp/mj OR **'breast conservation'**:ab,ti OR **'breast conservation therapy'**/exp/mj OR **'lumpectomy'**:ab,ti OR **'partial mastectomy'**:ab,ti	24,858	
3	**'mastectomy'**:ab,ti OR **'mastectomy'**/exp/mj	50,308	
4	**'quality of life'**:ab,ti OR **'quality of life'**/exp/mj OR **'wellbeing'**:ab,ti OR **'wellbeing'**/exp/mj	803,836	
5	#2 OR #3	61,247	
6	#1 AND #4 AND #5	1,103	2000 – 2025.

Search strategy for APA PsycArticles: 04.12.2024

**Search no**	**String**	**Results**	**Notes**
1	summary(cancer survivors) OR mjsub(cancer survivors) OR title(cancer survivors) OR summary(cancer patients) OR mjsub(cancer patients) OR title(cancer patients) OR summary(breast cancer patients) OR mjsub(breast cancer patients) OR title(breast cancer patients)	712	
2	summary(breast-conserving surgery) OR	10	

	mjsub(breast-conserving surgery) OR title(breast-conserving surgery) OR summary(breast conservation) OR mjsub(breast conservation therapy) OR title(breast conservation) OR summary(lumpectomy) OR mjsub(lumpectomy) OR title(lumpectomy)		
3	summary(mastectomy) OR mjsub(mastectomy) OR title(mastectomy)	41	
4	summary(quality of life) OR mjsub(quality of life) OR title(quality of life) OR summary(well-being) OR mjsub(well-being) OR title(well-being)	8,360	
5	#2 OR #3	41	
6	#1 AND #4 AND #5	9	

Search strategy for SciELO: 05.12.2024. excluded from the search

Search no	String	Results	Notes
1	(ab:(*cancer survivors)) OR (ti:(cancer survivors)) OR (ti:(cancer patients)) OR (ab:(cancer patients)) OR (ti:(breast cancer patients)) OR (ab:(breast cancer patients))	11602	
2	(ti:(breast-conserving surgery)) OR (ab:(breast-conserving surgery)) OR (ti:(breast conserving surgery)) OR (ab:(breast conserving surgery)) OR (ti:(breast conservation)) OR (ab:(breast conservation)) OR (ti:(lumpectomy)) OR (ab:(lumpectomy))	209	
3	(ti:(mastectomy)) OR (ab:(mastectomy))	627	
4	(ti:(quality of life)) OR (ab:(quality of life)) OR (ti:(well-being)) OR (ab:(well-being))	42163	
5	#2 OR #3	797	
6	#1 AND #4 AND #5	1	

Search strategy for VHL Regional portal: 05.12.2024. excluded from the search

Search no	String	Results	Notes
1	(cancer survivors) OR (cancer patients) OR (breast cancer patients)	58,965	search in title, abstract, subject
2	(breast-conserving surgery) OR (breast conserving surgery) OR (breast conservation) OR (lumpectomy)	805	
3	mastectomy	3178	
4	(quality of life) OR (well-being)	95,107	
5	#2 OR #3	3178	
6	#1 AND #4 AND #5	3116	2000-2025

Search strategy for Global Index Medicus: 05.12.2024. excluded from the search

Search no	String	Results	Notes
1	(cancer survivors) OR (cancer patients) OR (breast cancer patients)	97,984	search in title, abstract, subject
2	(breast-conserving surgery) OR (breast conserving surgery) OR (breast conservation) OR (lumpectomy)	1,536	
3	mastectomy	4,450	
4	(quality of life) OR (well-being)	81,960	
5	#2 OR #3	3,823	
6	#1 AND #4 AND #5	271	2000-2025
